# The Leicester, Leicestershire and Rutland quality improvement project and integrated chronic kidney disease system: implementation within a primary care network

**DOI:** 10.1186/s12882-024-03668-x

**Published:** 2024-08-08

**Authors:** Fahad Rizvi, Niraj Lakhani, Lydia Omuri, Simran Roshan, Tariq Kapasi, Samuel J. White, Philippe B. Wilson

**Affiliations:** 1 NHS Willows Health, 184 Coleman Road, Leicester, LE5 9LJ UK; 2 University Hospitals Leicester, Leicester Royal Infirmary, Infirmary Square, Leicester, Leicestershire, LE1 5WW UK; 3 School of Science and Technology, Clifton Campus, Nottingham Trent University, Nottingham, NG11 8NS UK; 4 York St John University, York, YO31 7EX UK

**Keywords:** Chronic kidney disease, Medicines optimisation, Clinical audit, Renal, Nephrology, Primary care

## Abstract

**Background:**

The optimisation of patients in primary care is a prime opportunity to manage patient care within the community and reduce the burden of referrals on secondary care. This paper presents a quality improvement clinical programme taking place within an NHS Primary Care Network as part of the wider Leicester Leicestershire Rutland integrated chronic kidney disease programme.

**Method:**

Patients are optimised to guidelines from the National Institute for Health and Care Excellence, by a primary care clinical team who are supported by nephrology consultants and nephrology pharmacists. Multidisciplinary team meetings take place with secondary care specialists and primary care staff. Learning is passed to the community clinicians for better patient treatment locally.

**Results:**

A total of 526 patients were reviewed under this project.The total number of referrals to secondary care which were discharged following first outpatient appointment, reduced from 42.9% to 10%. This reduction of 32.9% represents the optimisation of patient cases through this quality improvement project. Patients can be optimised and managed within the community, reducing the number of unnecessary referrals to secondary care.

**Conclusion:**

This programme has the potential to offer significant improvement in patient outcomes when expanded to a larger patient base. Medicine management and the use of clinical staff are optimised in both primary and secondary care.

## Introduction

Chronic kidney disease (CKD) is defined as a reduction in kidney function or structural damage (or both) present for more than 3 months [1]. It is associated with multiple complications such as acute kidney injury, endstage kidney disease (ESKD), and cardiovascular diseases. CKD is not a standalone condition that occurs in a patient; its aetiology stems from a host of other chronic diseases a patient may suffer from such as diabetes mellitus and hypertension [2]. Furthermore, there are several environmental and societal factors which can catalyse CKD and associated conditions. This includes demographics, health inequalities, genetic, cultural and biological contributors [3]. Combined with these comorbidities, CKD significantly contributes to increased mortality in patients. Therefore, the health implications to the health service for treating CKD appropriately are manifest [4]. Indeed, according to a recent report by Kidney Research UK, in 2023 kidney diseases account for approximately 3.2% of total NHS spending, representing £6.4bn. With CKD affecting more than 10% of the UK population, this figure is increasing proportionately with average population age [5].

For the optimal management of CKD there should be an aim to move away from treating standalone cardiovascular, kidney or metabolic pathologies and instead optimise treatment of these individuals in context of their multiple morbidities [6]. CKD is diagnosed by measuring the Estimated Glomerular Filtration rate (eGFR) and other factors such as proteinuria and haematuria. It is classified in 5 stages based on the eGFR and Albumin Creatine ratio (ACR) [7].

CKD Stage 1: eGFR > 90 ml/min/1.73m^2^,

CKD Stage 2: eGFR is 60-89 ml/min/1.73m^2^,

CKD Stage 3a: eGFR is 45-59 ml/min/1.73m^2^,

CKD Stage 3b: eGFR is 30-44 ml/min/1.73m^2^,

CKD Stage 4: eGFR is 15-29 ml/min/1.73m^2^,

CKD stage 5: eGFR is < 15 ml/min/1.73m^2^.

Each of the above 5 stages is further categorised by ACR levels with results given as a stage from 1 to 3:

A1 – an ACR of less than 3 mg/mmol.

A2 – an ACR of 3 to 30 mg/mmol.

A3 – an ACR of more than 30 mg/mmol.

The incidence and prevalence of CKD varies depending on the population studied, including ethnic group and socio-economic class. Kidney Research UK estimates that 7.2 million people currently have CKD in stages 1–5, equating to more than 10% of the population [8]. Public Health England produced a CKD prevalence model in 2014 using The Health Survey for England (HSE) 2010 report to show a clear association between increasing age and higher CKD stage 3a-5 prevalence [9]. It is likely that prevalence is rising as the population is ageing and several CKD risk factors such as obesity, type 2 diabetes, and hypertension are also increasingly common.

Currently, CKD is managed via a combination of tighter blood pressure (BP) control, lipid management, and lifestyle interventions (such as dietary advice, smoke cessation, and exercise). Medical treatment is usually administered in the form of Angiotensin-Converting Enzyme inhibitors (ACEi) or Angiotensin Receptor Blockers (ARBs) for cardiovascular and renal protection, and BP control, along with a statin for lipid control and reduction of cardiovascular disease risk [10]. In 2020, the DapaCKD study by Heerspink, et al. demonstrated positive outcomes with the use of Dapagliflozin in patients with CKD (irrespective of diabetes status). The study demonstrated “the risk of a composite of a sustained decline in the estimated GFR of at least 50%,’ and ‘end-stage kidney disease, or death from renal or cardiovascular causes significantly lower with dapagliflozin than with placebo” [11]. Based on this, in November 2021, the National Institute for Health and Care Excellence (NICE) recommended the use of Dapagliflozin for people with CKD and in March 2022 released Technology appraisal guidance (TA775). This provided evidence-based recommendations on its use [12]. Optimising the management of chronic kidney disease may improve patient outcomes, such as slowing the progression of the disease and reducing the risk of developing complications, such as cardiovascular disease [13].

Kidney Care UK estimate that around 3.5 m million people in the UK have the later stages of CKD (stages 3–5) with 30,000 people on dialysis. This number is rising as it is estimated that over 20 people develop kidney disease each day, with around 7,000 people waiting for a kidney transplant [14].

Complications from CKD arise from several well-known and newly understood mechanisms. Improvements in the understanding of these mechanisms have resulted in better optimisation of older therapies as well as the utilisation of novel treatments. These mechanisms include, increased neuro-hormonal activation, oxidative stress, inflammation, and platelet dysfunction, as well as phosphate retention leading to vascular calcification. In particular areas of neuro-hormonal activation, platelet dysfunction and inflammation have been increasingly studied and understood.

For patients with chronic heart failure as well as CKD, ACEi, and ARB therapies have shown inconsistent responses and bathe use of Angiotensin receptor and Neprilyn inhibitors have provided added benefits in by counteracting neurohormonal mechaniosms and reducing inflammation. The PARADIGM-HF and the PARAGON-HF studies both show the benefit of this new class of drifts on cardiovascular deaths and heart failure admissions.

Furthermore, SGLT2 inhibitors have been shown to have benefit from reducing platelet activation and enhancing sodium excretion. These effects have also translated to better cardiovascular outcomes and NICE guidelines have reflected on these benefits by including them in CKD management guidelines.

Glucan like peptide inhibitors as well as novel dual inhibitor drugs, such as tirzepatide, have also shown beneficial cardiovascular outcomes in patients with CKD.

Newer mineralocorticoid receptors antagonists, such as finerenone, have more beneficial effects as well as better adverse effect profiles than the older spironolactone. Given the advances in CKD management, as well as better cardiovascular outcomes, it is important that patients with CKD are managed by primary care teams with good support from Nephrology specialists as part of a MDT so that optimal therapies can be utilised early and monitored carefully in primary care [15].

It therefore follows that there may be a positive ripple effect from tackling CKD with optimising therapy as it may also have an impact on the cost burden of cardiovascular diseases and KRT for the NHS [16]. The 2021 UK Renal Registry Annual Report highlighted an increasing number of people on KRT, growing from 68,000 in 2020 to 69,500 in 2021 [17]. Furthermore, uncertainty surrounding appropriate referrals to secondary care is a recognised problem in healthcare potentially due to a clinician’s lack of confidence in their expertise about the most appropriate type of healthcare [18].

Given the significance of appropriate CKD management, the Willows Health CKD taskforce set out a novel quality improvement (QI) initiative designed to utilise multidisciplinary team (MDT) interventions and integrated digital systems aimed at improving kidney health for all. The novelty of this project lies within the combined use of educational outreach from secondary care consultants and the development of a series of decision-support and clinical management tools within the PCNs clinical management system, SystmOne to optimise CKD management. This may provide a replicable method of quality improvement for other PCNs.

This quality improvement project sought to improve CKD management and health outcomes, reduce unnecessary referrals to secondary care, and promote integration, maximising productivity and value for money across the healthcare system. Medicines optimisation in patients with CKD was another aim in this project, to improve patient health and outcomes.

This paper presents the results of a quality improvement project aimed at optimising the management of chronic kidney disease in primary care. The service redesign method, and participants in this quality improvement project will be outlined. Additionally, the development of the integrated digital system for this service redesign will be delineated. Furthermore, the impact of this service redesign, particularly in relation to referrals to secondary care, will be analysed and discussed.

## Aim

### The willows health CKD QI initiative

The aims of the QI initiative were to evaluate the current practice, where patients are not yet optimised with ACEi/ARBs and statins, and to facilitate the optimisation of CKD management in the PCN and where indicated, initiate the patient on Dapagliflozin. The legacy aims of the QI initiative were to improve knowledge and understanding amongst clinicians and patients around CKD, improve current practice and treatment of CKD and reduce unnecessary referrals to secondary care. Further aims include encompassing the management of CKD routinely with other comorbidities, and optimising therapy where possible in line with NICE TA775.

### Participants

## What is the patient demographic and how are patients identified as “at risk”

The Willows PCN has a wide and diverse demographic, with a majority of patients identifying as Asian and 23% of its patients living in deprived areas (Source: M&L CSU, NHS).

Patients are identified by two means. Firstly, through SystmOne searches. The second identification means is the use of The Kidney Failure Risk Equation (KFRE) [19] and urinary albumin to creatinine ratio (ACR) or eGFR to identify those patients at risk.

### Inclusion criteria

- All patients in the PCN living with CKD

### Exclusion criteria

- Patients deemed as extremely frail by the Clinical Pharmacist (anyone with a rockwood frailty score of 7 or greater)
- Patients already under specialist care
- Patients receiving dialysis
- Patients with a previous kidney transplant

## Method

### How are cases considered?

Patient cases are initially brought to CKD clinical sessions where a consultant nephrologist and nephrologist pharmacist discuss cases with the primary care team in a virtual MDT.

In the first MDT session, the primary care team consisting of the lead clinical pharmacist and support staff brought forward a range of patients.

Following the MDT, it was clear that significant multidisciplinary learning had taken place and the primary care team understood that many of these patients were not in fact complex and could be managed in the community with medicine optimisation.

These learnings led to optimisation clinics in the primary care network led by the clinical pharmacist where ACE inhibitor ( Angiotensin-converting-enzyme inhibitors) and STLG2 (Sodium-glucose co-transporter-2) dosage was optimised, leading to improved local management.

For this quality improvement project, the Model for Improvement, Plan Do Study Act cycle was utilised, as well as other aspects of healthcare quality improvement methods [20].

The model for improvement centres on three questions, which develop a clear aim, measures of success and confirm the desired change in healthcare. Following this, there are four stages:

Plan: This is where the quality improvement change will be planned, and the project plan developed.

Do: In this stage, the change will be implemented and tested.

Study: Here, data will be collected on the effects of the quality improvement implementation, using pre-agreed measurable outcomes. This data will then be analysed to examine the impact of the quality improvement change.

Act: Based on the previous stage, change can be fully implemented, or another plan for quality improvement will be made.

This model was the chosen method of improvement as it has been widely cited in health quality improvement and provides a structured approach, applicable to the primary care setting [21].

Furthermore, educational outreach, evidence-based guideline adoption and point-of-care reminders were employed in this project. These approaches were chosen as they have been reported to bring about the strongest effect in healthcare quality improvement [22].

1. The first stage of the quality improvement project included an evaluation of the current management of chronic kidney disease in the PCN. This allowed for the identification of challenges in the PCN’s management of chronic kidney disease that needed to be addressed.
2. Healthcare professionals in the primary care team then identified their cohort of patients with chronic kidney disease. A list of these patients was prepared for the multi-disciplinary sessions.
3. Following this, a multi-disciplinary session was organised. A consultant nephrologist, a nephrology pharmacist, and the primary care team would attend these sessions. The primary aim of these sessions was to collaboratively review the identified patients, using the NICE guidelines. The sessions also aimed to facilitate the education and training of the primary care team on chronic kidney disease medicine management.
4. After the completion of the multi-disciplinary session, the primary care team developed a decision support tool within SystmOne. The education from the multidisciplinary session, as well as information from the NICE guidelines was employed in the generation of this digital tool. Here, the SystmOne integrated support tool facilitated patient identification. The digital decision support tool identified patients who may have undiagnosed CKD and patients who may benefit from SGLT2i based on local CKD or NICE NG28 T2DM guidelines. Further explanation of this decision support tool is described in the discussion section.
5. Optimisation clinics were then organised in the PCN. These clinics were led by the clinical pharmacist and focused on utilising learning from the multi-disciplinary session to optimise ACE inhibitor and STLG2 dosage. During these clinics, patient with CKD stages 1 – 5 were identified. The HCPs then analysed these patients’ health statuses and complied patient data metrics. This included the patients’ hypertension status/treatment, their latest eGFR, ACR and diabetic status.
6. Thereafter, patient action plans were formulated. The action plans included the optimisation of BP and ACEi/ARBs if indicated. The decision on whether to initiate the patient on Dapagliflozin was also made.
7. The final stage of the quality improvement was to book the patients into specialist cardiovascular-renal-metabolic (CRVM) clinics. Here, the patients’ action plans would be carried out, ensuring that appropriate management and follow-up appointments were in place. In the case of more complex patients or uncertainty around the appropriate management of a patient, an integrated renal MDT between primary and secondary care clinicians was organised for this to be discussed.

### Measures

To gain an insight into the impact of this intervention, the following recorded outcomes were chosen:

- The number of patients referred to secondary care
- The number of patients optimised to the maximum tolerated RAASi [Renin Angiotensin Aldosterone inhibitor] therapy
- The number of patients optimised to NICE guidelines
- Patients’ comorbidities
- The number of patient with improved blood pressure

### Rationale for choosing these datapoints

The above measures were chosen as they were specific to the aim of this CKD quality improvement interventions. For instance, one of the aims of this project was to reduce unnecessary CKD referrals to secondary care. Therefore, the number of patient referrals to secondary care for CKD was recorded.

Furthermore, the patient intervention centered on optimising patients with CKD to the updated NICE guidelines. This provides the rationale for measuring the number of patients optimised to these guidelines, and to different therapies as a result of this optimisation.

Improved patient outcomes, specifically reduced blood pressure, was another measure chosen for this project. This measure was chosen as research indicates that improved blood pressure can slow the progression of CKD [23]. To acknowledge that improved patient outcomes may not be a direct result of this quality improvement project, all patients’ comorbidities were recorded. It was important to measure any improved patient outcomes, as they may have been as a result of the quality improvement project.

Patients' comorbidities were reviewed and recorded. Therefore, it is acknowledged that patients may have been on therapies for these comorbidities alongside the CKD optimisation. This may have affected patient outcomes during this project. However, the CKD homepage integrated into SystmOne, where additional comorbidities were managed. Subsequently, it was clear which patients were receiving therapies for their comorbidities alongside this intervention. The methods in place to ensure completeness and accuracy of data included using computerised data searches with SystmOne to measure patient datapoints.

### Analysis

Descriptive statistical analysis was employed for this quality improvement project.

## Results

**Fig. 1:**
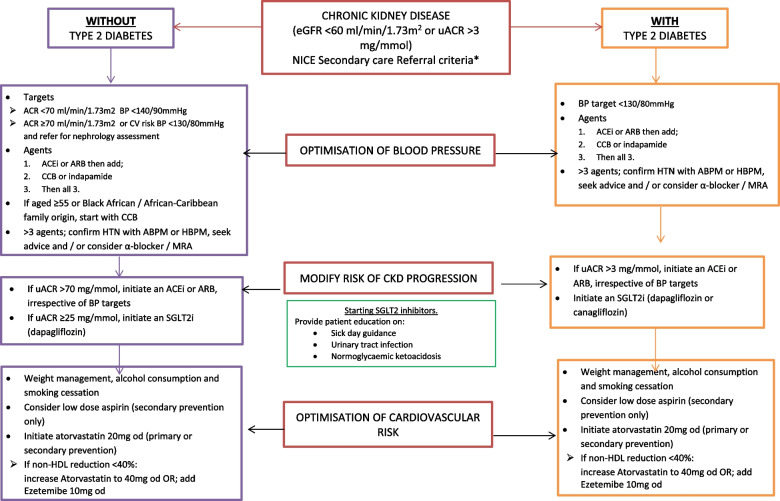
Medicines optimisation pathway and outline

A total of 526 patients were reviewed under this quality improvement project within the Willows PCN in Leicester. Of these, 407 were undergoing treatment for diabetes mellitus, 18 heart failure, and 101 were classified as under treatment for CKD alone with no associated complications or additional morbidities. Of the 526 patients reviewed, 371 were eligible for interventions. The remaining patients were either unsuitable for optimisation due to frailty, were under specialist care, or declined intervention. 329 patients were then optimised according to NICE guidelines (Fig. 1) with 298 having their blood pressure (BP) brought within target range. The remaining patients were not at target range but managed to their lowest tolerated BP. A total of 339 patients were optimised to the maximum tolerated RAASi therapy, where the remainder of patients were deemed unsuitable for RAASi according to NICE guidelines. Of the 329 patients who were optimised according to NICE guidelines, 100% tolerated and were optimised to addition of SGLT2i (Table 1).

**Table 1:** Summary of patient numbers within the quality improvement framework undertaken where DM: diabetes mellitus, HF: heart failure

Number of patients reviewed under CKD quality improvement initiative	526	%
With DM	407	77.7%
With HF	18	3.4%
With CKD alone	101	19.2%
Interventions	371	70.5%
Number of patients who had optimisation of treatment as per NICE guidelines	329	62.5%
BP bought in target range	298	56.7%
Max tolerated RAASi therapy	339	64.4%
Addition of SGLT2i	329	62.5%

Indeed, whilst the percentage of OP discharged at first attendance in secondary care nephrology clinics was 42.9% in 2021/22; this has reduced to 10% in 2022/23. This reduction of 32.9% can be attributed to improved management of patients in our primary care setting, improved nephrology medicines management and learning by the primary care clinical team and thereby only more complex and less traditional cases being referred to secondary care. This demonstrates that the MDT support and nephrology pharmacist support can lead to improved patient care in the community, whilst reducing the burden of unmanaged and unoptimized referrals to secondary care.

### NICE referral criteria

Taking into account the individual's wishes and other health conditions, considering referral to a hospital kidney doctor if:

- 5-year KFRE predicted risk over 5%

Other NICE referral criteria include:

- an ACR of 70 mg/mmol or more, unless known to be caused by diabetes and already appropriately treated
- ACR of more than 30 mg/mmol (ACR category A3), together with haematuria
- a sustained decrease in eGFR of 25% or more and a change in eGFR category within 12 months
- a sustained decrease in eGFR of 15 ml/min/1.73 m2 or more per year
- hypertension that remains poorly controlled (above the person's individual target) despite the use of at least 4 antihypertensive medicines at therapeutic doses
- known or suspected rare or genetic causes of CKD
- suspected renal artery stenosis.

https://www.nice.org.uk/guidance/ng203/chapter/Recommendations#risk-assessment-referral-criteria-and-shared-care

## Discussion

The impact of this quality improvement project resulted in the reduction of unnecessary referrals to secondary care and provided a standardised approach for managing patients with CKD in primary care.

There are several strengths to the implementation of this project. Most poignantly, the project allowed for the adherence to NICE recommended guidelines, ensuring that every effort is made to optimise patients with CKD to these guidelines. Moreover, this project’s use of educational multi-disciplinary meetings facilitated the upskilling of NHS staff. In addition to this, the use of the Model for Improvement in this project allows for replication in other primary care networks.

### Secondary care educational outreach impact

MDT meetings were initially scheduled for every 8 weeks with patient cases being considered at each meeting. However, after the initial MDT where collaborative learning took place between the secondary and primary care teams, patients are now initially managed within the PCN through ACE and STLG2 optimisation, with only complex patients being referred to secondary care. The turnaround of patient cases has now reduced to 2 weeks from internal referral to a medicines optimisation with a clinical pharmacist within the PCN team. The confidence and experience in the clinical team within the PCN has now been sufficiently upskilled to see a significant reduction in cases referred to secondary care. Indeed, from a cohort of 526 patients where previously 30 patients were being referred to secondary consults each month, this has now reduced to 2.

The multidisciplinary meetings allowed for learning to be passed on to the primary care clinicians and community members. The team education provided the primary care clinicians with the confidence to manage patients with chronic kidney disease without the need of secondary care referral. Easy to obtain advice and guidance and case discussions with secondary care consultants has improved CKD understanding and management amongst primary care clinicians and their teams. Furthermore, the limited time requirement by primary and secondary care staff to work within the MDT is economically mitigated by the time saving of the improved optimisation of patients in the community, as well as the development and implementation of the digital tools and case forms described herein.

### Challenges from the previous pathway of care that have been addressed by the QI project

The quality improvement project has transformed the pathway of care for managing patient with chronic kidney disease in the PCN. Prior to review in CVRM clinics, patients were rarely optimised to gold-standard therapies with the correct polypharmacy and target cardio-metabolic readings. Of the 526 patients in the project, only approximately 20% of patients were already optimised to the stage where Dapagliflozin would be the only step in medicines optimisation. All other patients were in need of multiple levels of optimisation and were therefore booked in to enhanced CVRM clinics to facilitate this.

### Implementation into PCN digital health services

Based on the tools and learning developed through application of the iCKD programme within the PCN, the pharmacy team was able to design and implement a series of decision-support and clinical management tools within the PCNs clinical management system, SystmOne.

### Case finding and patient identification

This consists of two elements: (i) the identification of patients with potentially undiagnosed CKD, and (ii) identifying patients who would benefit from SGLT2i based on local CKD or NICE NG28 T2DM guidelines.

**Fig. 2:**
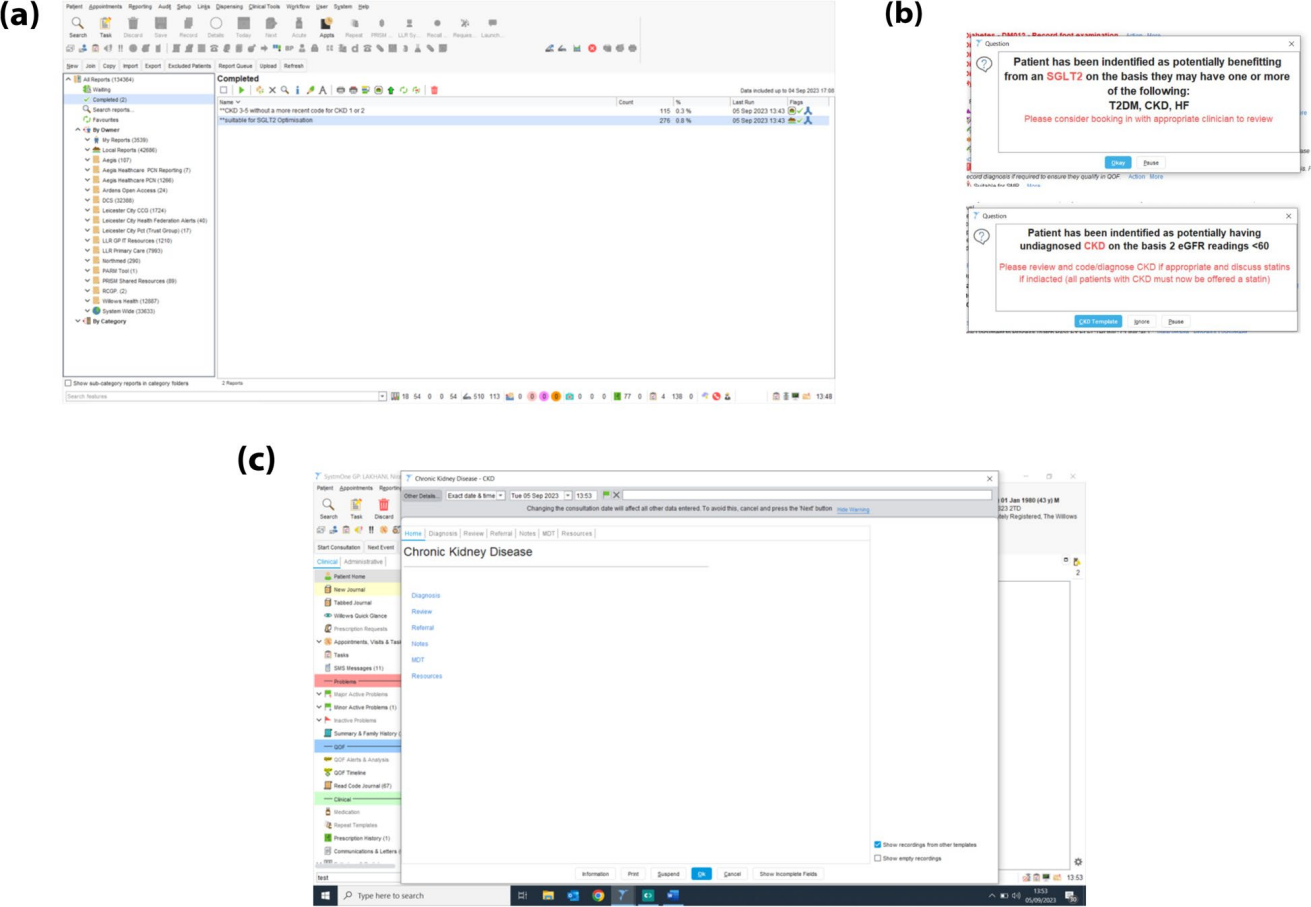
(**a**) Screenshot of searches automated and completed for both identification of undiagnosed CKD and potential for medicines optimisation. (**b**) Pop-up messages automatically applied to the patient files once search has been undertaken which guide the clinician to follow the prompts to the diagnosis page. (**c**) CKD Homepage within SystmOne allows for optimised follow-up through the clinical pathway

Once patients are identified through these searches (Fig. 2a), their record has a pop-up message attached when opened which prompts the clinician based on the results of the search (Fig. 2b).

A CKD Home Page has been created for clinicians, which facilitates their decision making and parsing of clinical records through each stage of the CKD care pathway. Sections are attributed to diagnosis, review, referral, additional notes and MDT, as well as a useful resources section (Fig. 2c).

**Fig. 3:**
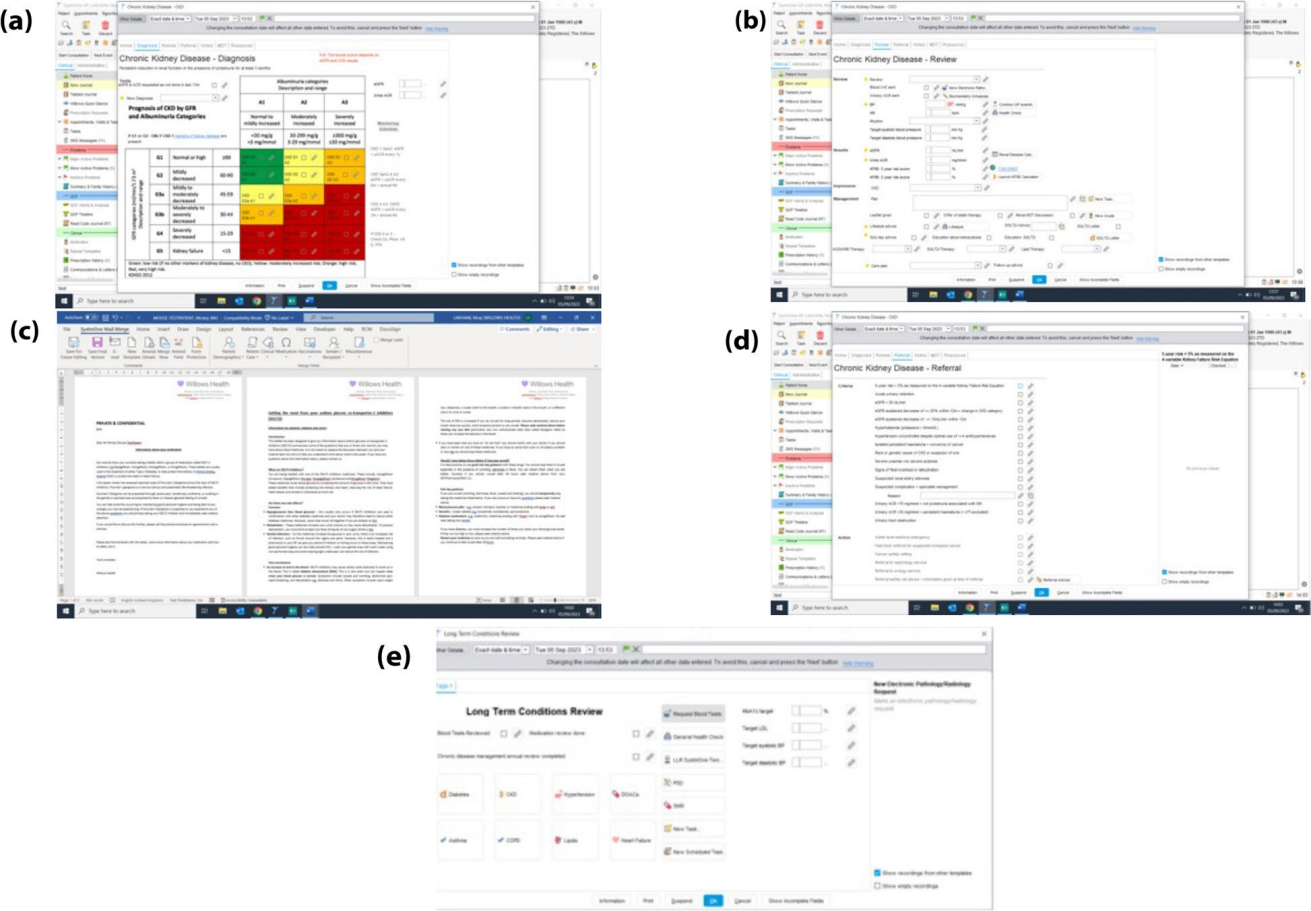
(**a**) KDIGO heatmap based diagnostic matrix for CKD patients implemented within the SystmOne CKD portal designed within the PCN. (**b**) The CKD review page, which supports the monitoring of the clinical case including single click functionality for calculating such functions as KFRE. (**c**) Automated letter created from the SystemOne CKD instance and tailored to the patient case for their medications and CKD stage. (**d**) Referral criteria are outlined in a manner which is easy to access and implement for the clinician whilst ensuring that referrals are only carried out for patients who have not been successfully optimised or are inappropriate for such optimisations. (**e**) Chronic disease review template allows for more holistic reviews of conditions such as CKD, which is incorporated into the wider chronic clinical management framework

The diagnosis tab is based on the KDIGO head map [24] which allows for early diagnosis and prompt management of the condition (Fig. 3).

Once diagnosis has been completed, a review page supports monitoring by automatically calculating KFRE through a single click-through option (Fig. 3b). This allows for seamless data gathering and capturing additional clinical data for these patients within the pathway. It also enables factors such as medication and lifestyle to be included and codes for these within SystmOne appropriately. Currently, we include: Lifestyle advice, ACEi/ARB optimisation, SGLT2 optimisation, and Lipid management.

Additionally, the template also allows the clinician to code any leave guidance such as sick days which have been recommended, as well as DKA and SGLT2 education and allows for the production of patient leaflets and letters in an automated fashion and improves the safety of prescribing such medications (Fig. 3c).

Referral criteria (Fig. 3d) within the “Referral” tab of the CKD Homepage on SystmOne have been designed to (a) aid the clinician in ensuring the necessity of referral is fully justified and (b) highlighting that optimisation and other support measures have either been unsuccessful or inappropriate for the specific case within the PCN and thereby necessitating support from secondary care.

As part of the integration within the wider continuous management workstream, the CKD digital tools described herein are also included within our chronic disease management toolkit (Fig. 3e) which allows for more holistic review of such often complicated clinical cases.

Multi-disciplinary Meetings and CVRM Clinics.

The MDT meeting supported learning on CKD medication and management optimisation. Following this meeting, the lead clinical pharmacist in the PCN had the confidence to lead the CVRM clinic. Each patient attending the clinic was followed up shortly after the clinic and then again after 2–3 months to ensure there was no deterioration. The impacted the workflow for CKD management in the PCN in positive way, with more organisation and a clear agenda for each consultation with the patient. The incorporation of decision support tools in SystmOne integrated well within the PCN. SystmOne was a management tool used by all staff members, and the use of computerized reminders and alerts allowed for centralisation of CKD management in the PCN.

### Comparison with previous literature

Previous research has presented similar quality improvement interventions in primary care for CKD management. For example, the TRANSLATE study examined the implementation of evidence-based guidelines for CKD into primary care. This study was in response to primary care physician’s sub-optimal management and understanding of the early diagnosis of CKD. The study identified several barriers to implementing the guidelines, including limited time and challenges with using data to monitor progress [25]. Contrastingly, the quality improvement project presented in this paper mitigated some of these challenges as the evidence-based guideline implementation in this study was supported by computer decision support tools, which were integrated in and developed using the pre-existing electronic medical record, SystmOne. NHS staff were familiar with this EMR. Therefore, additional time for training on its use and data recording was not required. Additional studies have employed the use of computerised tools when improving CKD management in primary care. For instance, an observational controlled study in 2019 aimed to improve coding and CKD management in primary care by using education sessions, and computerised quality improvement tools. Improvements in patients’ blood pressure targets were identified as a result in this study [23].

## Limitations

This study is subject to certain limitations. Firstly, the number of patients included in this study may limit the generalisabilty of results. A larger sample size would enhance the study’s external validity and potentially provide a more comprehensive understanding of the impact of the interventions. Additionally, the duration of the study may present a limitation as observation of the effects of the intervention over a longer period of time may have been useful. A longer term follow up maybe be beneficial to capture the sustained effects of the intervention. However, further research on the effects of this intervention after an extended period of time may be completed, following this study.

## Conclusions

The MDT pathway has proven to be of great utility, by reduced unnecessary referrals into secondary care as well as expediting those who needed to be seen promptly. This initiative is an ongoing project but early data shows that there is a need for this type of activity across early nephrology care.

There is the potential for the findings of this project to be utilised in other contexts. The use of decision support tools developed using the pre-existing patient management system, SystmOne, proved to facilitate the quality improvement project as the interventions fit into pre-established pathways of care. This highlights that developing digital interventions that intergrate with pre-exisiting digital systems may assist with quality improvement projects in other areas of healthcare.

Similarly, the utilisation of the educational outreach to secondary care consultants encouraged the upskilling of staff in this project and contributed to the reduction of unnecessary secondary care referrals. This is a method of quality improvement that has the potential to be employed in different areas of healthcare.

This early data is highly promising and demonstrates the need to see this QI project further develop and to ensure there is a legacy effect in place for future CKD patients. If our initial findings are representative of CKD management nationally, further work and roll out of such QI initiatives will be of significant importance. Indeed, such capabilities as outlined herein are now available to all primary care networks within the LLR region.

## Data Availability

The datasets generated and/or analysed during the current study are available from the corresponding author on reasonable request.

## Abbreviations

CKD: Chronic Kidney Disease
QI: Quality Improvement
NICE: National Institute for Health and Care Excellence
ESKD: Endstage Kidney Disease
eGFR: Estimated Glomerular Filtration rate
ACR: Albumin Creatine Ratio
HSE: Health Survey for England
KRT: Kidney Replacement Therapy
BP: Blood Pressure
ACEi: Angiotensin-Converting Enzyme Inhibitors
ARBs: Angiotensin Receptor Blockers
TA775: Technology appraisal guidance
CVRM: Cardiovascular-Renal-Metabolic
KFRE: The Kidney Failure Risk Equation
RAASi: Renin-angiotensin-aldosterone system inhibitors
DM: Diabetes Mellitus
HF: Heart Failure
SGLT2i: Sodium-glucose co-transporter-2 inhibitors
OP: Outpatient
uACR: Urine Albumin-Creatinine Ratio
CV: Cardiovascular
CCB: Calcium-Channel Blocker
HTN: Hypertension
ABPM: Ambulatory Blood Pressure Monitoring
HBPM: Home Blood Pressure Monitoring
MRA: Mineralocorticoid Receptor Antagonists
KDIGO: Kidney Disease Improving Global Outcomes
PCN: Primary Care Network
DKA: Diabetic Ketoacidosis
